# Elevated PD-L1 and PECAM-1 as Diagnostic Biomarkers of Acute Rejection in Lung Transplantation

**DOI:** 10.3389/ti.2024.13796

**Published:** 2024-11-21

**Authors:** Rene Novysedlak, Jan Balko, Janis Tavandzis, Vira Tovazhnianska, Antonij Slavcev, Katerina Vychytilova, Jitka Smetanova, Alexandre Bohyn, Jaromir Vajter, Martina Borcinova, Bart M. Vanaudenaerde, Robert Lischke, Jiri Vachtenheim, Laurens J. Ceulemans, Zuzana Ozaniak Strizova

**Affiliations:** ^1^ Prague Lung Transplant Program, 3rd Department of Surgery, First Faculty of Medicine, Charles University and Motol University Hospital, Prague, Czechia; ^2^ Laboratory of Respiratory Diseases and Thoracic Surgery (BREATHE), Department of Chronic Diseases and Metabolism, KU Leuven, Leuven, Belgium; ^3^ Department of Pathology and Molecular Medicine, Second Faculty of Medicine, Charles University and Motol University Hospital, Prague, Czechia; ^4^ Department of Immunogenetics, Institute for Clinical and Experimental Medicine, Prague, Czechia; ^5^ Department of Immunology, Second Faculty of Medicine, Charles University and Motol University Hospital, Prague, Czechia; ^6^ Department of Public Health and Primary Care, Leuven Biostatistics and Statistical Bioinformatics Center (L-BioStat), KU Leuven, Leuven, Belgium; ^7^ Department of Anesthesiology, Resuscitation and Intensive Care Medicine, Second Faculty of Medicine, Charles University and Motol University Hospital, Prague, Czechia; ^8^ Department of Obstetrics and Gynecology, First Faculty of Medicine, Charles University, General University Hospital, Prague, Czechia; ^9^ Department of Thoracic Surgery, University Hospitals Leuven, Leuven, Belgium

**Keywords:** lung transplantation, acute cellular rejection, immunohistochemistry, luminex, checkpoint inhibitors

## Abstract

Acute cellular rejection (ACR) frequently occurs following lung transplantation (LuTx) and represents a risk factor for the development of chronic lung allograft dysfunction (CLAD) as well as long-term survival. The histopathological diagnosis of ACR carries a burden of interobserver variability. The widespread utilization and cost-effectiveness of immunohistochemistry (IHC) was proven beneficial in diagnosing rejection in human kidney transplantations and LuTx rat models. However, its potential for ACR detection in patients remains unexplored. We analyzed surface markers (CD3, CD4, CD8, CD20, CD68, CD47, PD-1, PD-L1, and CD31/PECAM-1) on lung tissue cryobiopsy samples collected within 6 months post-LuTx from 60 LuTx recipients, 48 of whom were diagnosed with ACR. Additionally, serum samples from 51 patients were analyzed using a multiplex bead-based Luminex assay. The cytokines and markers included PD-L1, IL2, TNFα, IFNγ, and Granzyme B. We observed a significant increase in PD-L1 tissue expression within the rejection group, suggesting a concerted effort to suppress immune responses, especially those mediated by T-cells. Furthermore, we noted significant differences in PECAM-1 levels between ACR/non-ACR. Additionally, peripheral blood C-reactive-protein levels tended to be higher in the ACR group, while Luminex serum analyses did not reveal any significant differences between groups. In conclusion, our findings suggest the potential value of PECAM-1 and PD-L1 markers in diagnosing ACR.

## Graphical Abstract

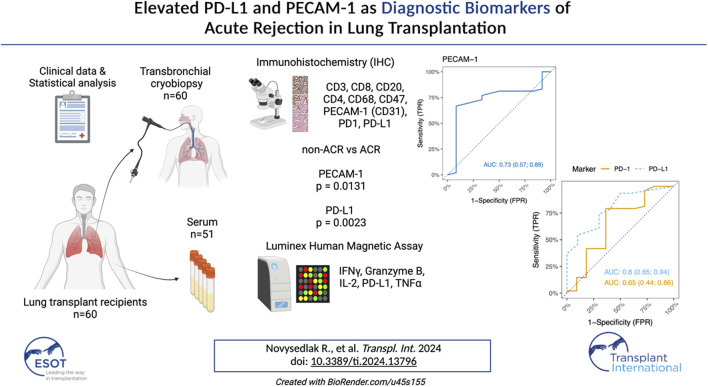

## Introduction

Long-term allograft survival has always been significantly challenged by the persistent risk of transplantation rejection [1–4]. During transplantation, both ischemia-reperfusion and mechanical injury as well as inadequate organ storage conditions prompt immune system reactions through the local release of cytokines, chemokines, adhesion molecules, damage-associated molecular patterns, and other signaling molecules [5–7]. These events trigger an influx of innate immune cells to the graft, which is followed by the presentation of allogeneic antigens by antigen-presenting cells (APCs) to adaptive immune cells [8].

Acute organ rejection involves acute cellular rejection (ACR) orchestrated by T-cells and acute humoral rejection (AMR) driven by antibody-producing plasma cells [9].

Antibody-mediated rejection (AMR) is a profoundly studied phenomenon particularly in kidney transplants, leading to standardized nomenclature and diagnostic criteria. However, its applicability in lung transplants is limited, emphasizing the significance of T-cell-mediated rejection in lung allografts [10].

T-cell-mediated ACR in lung transplants, impacting small airways and vasculature, represents a significant clinical challenge [11–13]. The incidence of ACR is highest early post-lung transplantation, with 27% of adult patients experiencing at least one treated episode within the first year. ACR is associated with bronchiolitis obliterans syndrome (BOS), a main phenotype of chronic lung allograft dysfunction (CLAD), with late ACR episodes (after 180 days post-transplant) linked to an elevated risk of BOS [1, 14–18].

Diagnostic assessment of ACR faces interobserver variability, particularly in lower-grade rejection, and understanding of the specific traits and phenotypic patterns of infiltrating T-cells during ACR remains limited [11]. Therefore, ACR demands attention from researchers to pinpoint potential biomarkers that could help to understand immune responses and strengthen the diagnostic process and early detection of rejection.

Immune checkpoint molecules have been extensively studied in the oncological context [19]. However, their role and potential use in solid organ transplantation is far from being understood. Several studies have shown that the interaction between Programmed Death-Receptor 1 (PD-1) and Programmed Death-Ligand 1 (PD-L1) is essential for both initiating and sustaining tolerance to the graft [20].

PD-1 is a key inhibitory receptor involved in both adaptive and innate immune responses. It is expressed on various immune cells, including activated T cells, natural killer cells, B lymphocytes, macrophages, dendritic cells, and monocytes. PD-1 plays a crucial role in dampening autoimmune reactions and thus, preserving immune tolerance [21, 22]. As a PD-1 ligand, PD-L1 is typically found on macrophages, activated T and B cells, dendritic cells, and various epithelial cells, with its expression being elevated under inflammatory conditions. PD-L1 is often found in immune environments characterized by high loads of CD8^+^ T cells and the production of Th1 cytokines and interferons [21].

In contrast to other costimulatory molecules, PD-L1 expression extends beyond hematopoietic cells, as it can also be detected on endothelial cells, placental trophoblasts, and even pancreatic islet cells [23].

In the context of transplantation, the PD-1/PD-L1 pathway has been primarily investigated in animal models, with limited research was conducted in humans, particularly among lung transplant recipients [24, 25].

PD-L1 expression was shown to be significantly upregulated following transplantation on endothelial cells within heart allografts [26].

This increased expression within the vasculature indicates that PD-L1 may play a crucial role at the interface between immune cells and the transplanted organ, highlighting its potential importance in regulating the alloimmune response. In this regard, molecules involved in endothelial-immune cell interactions warrant particular attention.

Platelet/endothelial cell adhesion molecule-1 (PECAM-1 or CD31) is a key regulator of leukocyte transmigration across the endothelium and has been shown to be essential for transendothelial migration. PECAM-1-mediated leukocyte migration can be effectively inhibited by PECAM-1-specific blocking antibodies or by downregulating PECAM-1 expression [27].

Thus, examining PD-1/PD-L1 coinhibitory signals could provide valuable insights into the regulation of the alloimmune response, while clarifying the involvement of PECAM-1 in transplant rejection could highlight its potential as a novel therapeutic target in transplantation.

Immunohistochemistry (IHC), a cost-effective technique, has proven useful in diagnosing ACR in human kidney transplants [28–30]. Although in rat models, IHC aimed specifically at CD4⁺ and CD8⁺ T-cell proportions and distribution, improved the sensitivity and specificity of lung rejection diagnosis and grading, the same approach in human lung ACR is insufficient [31].

To address these gaps, and to better understand the role of immune checkpoint molecules in transplantation, our study explores multiple IHC biomarkers, including CD3, CD4, CD8, CD20, CD68, CD47, PD-1, PD-L1, and CD31/PECAM-1 within a large cohort. We aimed to identify T-cell subtype proportions and phenotypes, assess immune exhaustion levels, understand immune system dynamics, examine leukocyte transendothelial migration patterns, evaluate “don’t eat me” signals expression, and determine macrophage and B-cell proportions within lung allograft specimens.

Additionally, to obtain a detailed understanding of the immune landscape in LuTx recipients, we have extended our analyses by measuring T cell functionality via a multiplex assay. To provide a comprehensive profile of the immune status and functionality of T cells, which are critical in the context of transplantation, PD-L1, IL-2, Granzyme B, Tumor Necrosis Factor alpha (TNFα) and interferon gamma (IFNγ), were evaluated.

PD-L1 was included due to its role in immune regulation, whereas IL-2 provided insights into the activation status and responsiveness of T cells. Granzyme B, TNFα, and IFNγ are integral to the effector functions of T cells. Together, they provide comprehensive insights into T cells’ cytotoxic potential, inflammatory responses, and the regulatory balance of immune activation, all of which are crucial for graft survival and effective immune defense.

Understanding not just the phenotype but also the function of T cells is vital for developing strategies to enhance graft survival and reduce the risk of rejection.

This comprehensive analysis aimed to provide crucial insights into immune events within lung allografts.

## Materials and Methods

### Study Design

This retrospective study includes 171 adult patients (≥18 years) who underwent bilateral lung transplantation (LuTx) at Motol University Hospital in Prague between 1 January 2018, and 31 December 2021. Excluded were single, lobar, and multiorgan LuTx, as well as re-transplants. Patients without cryobiopsy within the initial 6 months post-transplant, lacking cryopreserved samples, or tissue samples for research were also excluded.

Routine and on-demand cryobiopsies were collectively analyzed, with routine samples taken after one, three, or six post-transplant months. Demographics and clinical data were obtained from patient files, and only laboratory results before ACR treatment initiation were considered. Serum samples taken prior to the initiation of potential rejection treatment were analyzed for selected cytokines using a customized Luminex Human Magnetic Assay. Tissue samples were evaluated using IHC. The study, approved by the Ethics Committee of Motol University Hospital (EK-530/21), received written informed consent from all patients at transplantation listing. Follow-up was censored on 24 September 2023.

### Study Population

Donor and preservation variables included: age, gender, weight, height, BMI, donor type [donation after brain death (DBD) vs. donation after circulatory death], cytomegalovirus (CMV) status and times of ischemia for both lungs.

Recipient variables included: age, gender, weight, height, BMI, CMV status, underlying comorbidities, indication for transplant, immunosuppression regimen used, date of the first post-transplant lung tissue cryobiopsy, acute cellular rejection (grades A and B), infection status, peripheral blood levels of C-reactive protein (mg/L), peripheral blood levels of white blood cells (×10^9^/L) and percentage and count (×10^9^/L) of its subtypes, namely, lymphocytes, monocytes, neutrophils, eosinophils, basophils and immature granulocytes.

### Immunohistochemistry

Sixty formalin-fixed paraffin-embedded tissue samples were retrospectively analyzed, evaluating the expression of CD3, CD8, CD20, CD4, CD68, CD47, PECAM-1 (CD31), PD1, and PD-L1. Histologic sections (3 µm thick) underwent staining with specific antibodies, including Anti-CD3, Anti-CD8, Anti-CD20, Anti-CD4, Anti-CD68, Anti-CD47, Anti-CD31, Anti-PD1, and Anti-PD-L1. Staining protocols involved various pre-treatments and dilutions (Table 1), with detection using a micropolymeric non-biotin system, except for PD-L1. Manual scoring by an experienced pathologist assessed the absolute count of immune cells positive for selected markers per 1 mm^2^, starting from “hot spots” within each sample. Our study focused on immune cells and evaluated cytoplasmic and membranous staining. Specifically, PECAM-1 showed membranous staining and PECAM-1-positive endothelial cells were excluded from scoring. Differentiation of PECAM-positive immune cells (specifically intraalveolar macrophages) from endothelial cells was enabled by distinct characteristics of the macrophages, such as their morphology, intra-alveolar location, and lower staining intensity, as shown in Figure 4. The nuclei were counterstained with hematoxylin.

**TABLE 1:** Immunohistochemistry staining specifications.

Antibody	Clone; manufacturer	Dilution	Pre-treatment
anti-CD3	RBT-CD3 [BioSB]	1:20	Heating in a buffer solution of pH9 in a water bath
anti-CD8	C8/144B [Dako]	1:200	Heating in a buffer solution of pH9 in a water bath
anti-CD20	L26 [Dako]	1:300	Heating in a buffer solution of pH6 in a water bath
anti-CD4	4B12 [BioGenex]	1:250	Heating in a buffer solution of pH9 in a water bath
anti-CD68	PG-M1 [Dako]	1:100	Heating in a buffer solution of pH9 in a water bath
anti-CD47	PA5-80435 [Thermofisher Scientific]	1:200	Heating in a buffer solution of pH6 in a water bath
anti-CD31 (anti-PECAM-1)	JC70A [Dako]	1:40	Heating in a buffer solution of pH6 in a water bath
anti-PD1	polyclonal serum [Abd Serotec]	1:200	Without antigen retrieval
anti-PD-L1	22C3 [Dako]	Certified kit	Processed according to the certified Autostainer Link 48 protocol

### Luminex Assay

Our sample preparation procedures were followed in accordance with the manufacturer’s guidelines to ensure accuracy and reproducibility. Specifically, we focused on blood sera derived from 51 patients and analyzed a panel of cytokines and markers, including IFNγ, Granzyme B, IL-2, PD-L1 and TNFα. A customized Luminex Human Magnetic Assay, sourced from Biotechne, R&D Systems s.r.o. in Prague, was used. The assay enabled precise detection of cytokines and chemokines in serum from lung transplant samples. Data were acquired using the Bio-Plex 200 system.

### Cryobiopsies

Transbronchial cryobiopsy was the method of lung tissue sample collection, facilitated through flexible bronchoscopy targeting primarily the left lower lobe when possible. This procedure, conducted under total anesthesia, adhered to standard medical protocols. Cryobiopsies were evaluated according to ISHLT guidelines and scored for acute cellular rejection (ACR) (Grade A) and lymphocytic bronchiolitis (Grade B) [32]. Both tissue samples and peripheral blood were meticulously preserved at a stable temperature of −80°C until the analysis was performed.

### Statistical Analyses

Continuous variables were standardly reported as median (interquartile range) and categorical variables as number (percentage). Data were grouped into two main groups – control group (only grade A0 = non-ACR) and rejection group (ACR grade A1-3). Fisher’s exact test was used to compare categorical variables between groups. Spearman correlations (ρ) and Mann–Whitney U tests were used to evaluate relations between clinical, IHC and Luminex variables and ACR. Kruskal-Wallis test was used to evaluate relations between all A0-A3 groups (Supplementary Table S1). Values falling below the lower limit of quantification were subjected to a halving procedure in the analytical process.

To evaluate the predictive capacity of IHC markers for graft acceptance or rejection (non-ACR vs. ACR), individual Receiver Operating Characteristic (ROC) curves were constructed for each marker, and the corresponding Area Under the Curve (AUC) was calculated. The Youden’s Index and Euclidian distance were computed to find the ideal cut-off values. This part of the analysis was performed by an experienced biostatistician (A.B.).

## Results

### Study Population and Baseline Characteristics

Immunohistochemical analyses were performed on a cohort of 60 tissue samples obtained from 60 bilateral lung transplant (LuTx) recipients. However, for subsequent Luminex analyses, samples from 9 patients were unavailable. Recipient age was 53 (42–60) years. Among recipients, 40 (67%) were male and 20 (33%) female. Indications for transplantation included chronic obstructive pulmonary disease (COPD) in 19 (32%) patients, interstitial lung disease (ILD) in 26 (43%), cystic fibrosis in 8 (13%) and pulmonary arterial hypertension (PAH) in 7 (12%) patients. Only DBD donors were reported in this cohort. Donor age was 44 (31–53) years. Two (3%) donors were older than 70 and one (2%) was younger than 18. Among donors, 32 (53%) were male, 28 (47%) female. Tables 2, 3 summarize the baseline characteristics of the study cohort. No differences in baseline characteristics were observed between control and rejection group. Supplementary Table S1 presents the distribution of acute cellular rejection grades in the study cohort. Standard induction immunosuppression at our center consists of basiliximab, tacrolimus, mycophenolate, and corticosteroids. For selected patients, an alternative strategy to basiliximab is employed. Supplementary Table S2 presents the percentage of patients in whom basiliximab and each alternative modality to it was used, either alone or in combination. For maintenance immunosuppression tacrolimus, mycophenolate, and corticosteroids are used. No significant difference was observed when comparing induction immunosuppressive regimens (*p* = 0.3341) and infection status (*p* = 0.7191) between the groups (Supplementary Tables S2, S3). A description of underlying immunological conditions is provided in Supplementary Table S4.

**TABLE 2:** Cohort donor, preservation, and recipient characteristics.

Cohort characteristics	Results
**Donor and preservation**	
Age at donation, years	44 (31–53)
*Sex, n (%)*	
Male	32 (53)
Female	28 (47)
Body mass index, kg/m^2^	24 (22–26)
*CMV status, n (%)*	
Positive	39 (65)
Negative	21 (35)
Total ischemic time (longest time of two lungs), min	345 (294–390)
Total ischemic time (mean of two lungs), min	293 (245–330)
**Recipient**	
Age at transplant, years	53 (43–60)
*Sex, n (%)*	
Male	40 (67)
Female	20 (33)
Body mass index, kg/m^2^	26 (19–28)
*Indication for transplant, n (%)*	
Chronic obstructive pulmonary disease	19 (32)
Interstitial lung disease	26 (43)
Cystic fibrosis	8 (13)
Pulmonary arterial hypertension	7 (12)
Time spent on waiting-list, days	183 (85–354)
*CMV status, n (%)*	
Positive	37 (62)
Negative	20 (33)

Data are expressed as median (interquartile range) if not otherwise indicated. Abbreviation: CMV, cytomegalovirus.

**TABLE 3:** Baseline characteristics of control and rejection group.

	no ACR (A0) n = 12	ACR (A1-3) n = 48	*p*-value
Donor age, years	45 (36–54)	43 (31–50)	0.54
Male donors	8	24	0.35
Female donors	4	24	
Donor BMI, kg/m^2^	24 (23–26)	24 (22–27)	1.0
Total ischemic time (longest time of two lungs), min	299 (281–364)	351 (304–396)	0.12
Total ischemic time (mean of two lungs), min	255 (239–314)	297 (256–331)	0.13
Recipient age, years	58 (48–61)	52 (38–59)	0.39
Male recipients	9	31	0.73
Female recipients	3	17	
Recipient BMI, kg/m^2^	26 (22–28)	26 (19–27)	0.64
*Indication for transplant*	—		
Chronic obstructive pulmonary disease	5	14	0.59
Interstitial lung disease	6	20	
Cystic fibrosis	1	7	
Pulmonary arterial hypertension	0	7	

Data are expressed as simple count (categorical variables) or median (range), respectively. Abbreviation: BMI, body mass index.

### The Levels of C-Reactive Protein Were Associated With Acute Cellular Rejection

**FIGURE 1:**
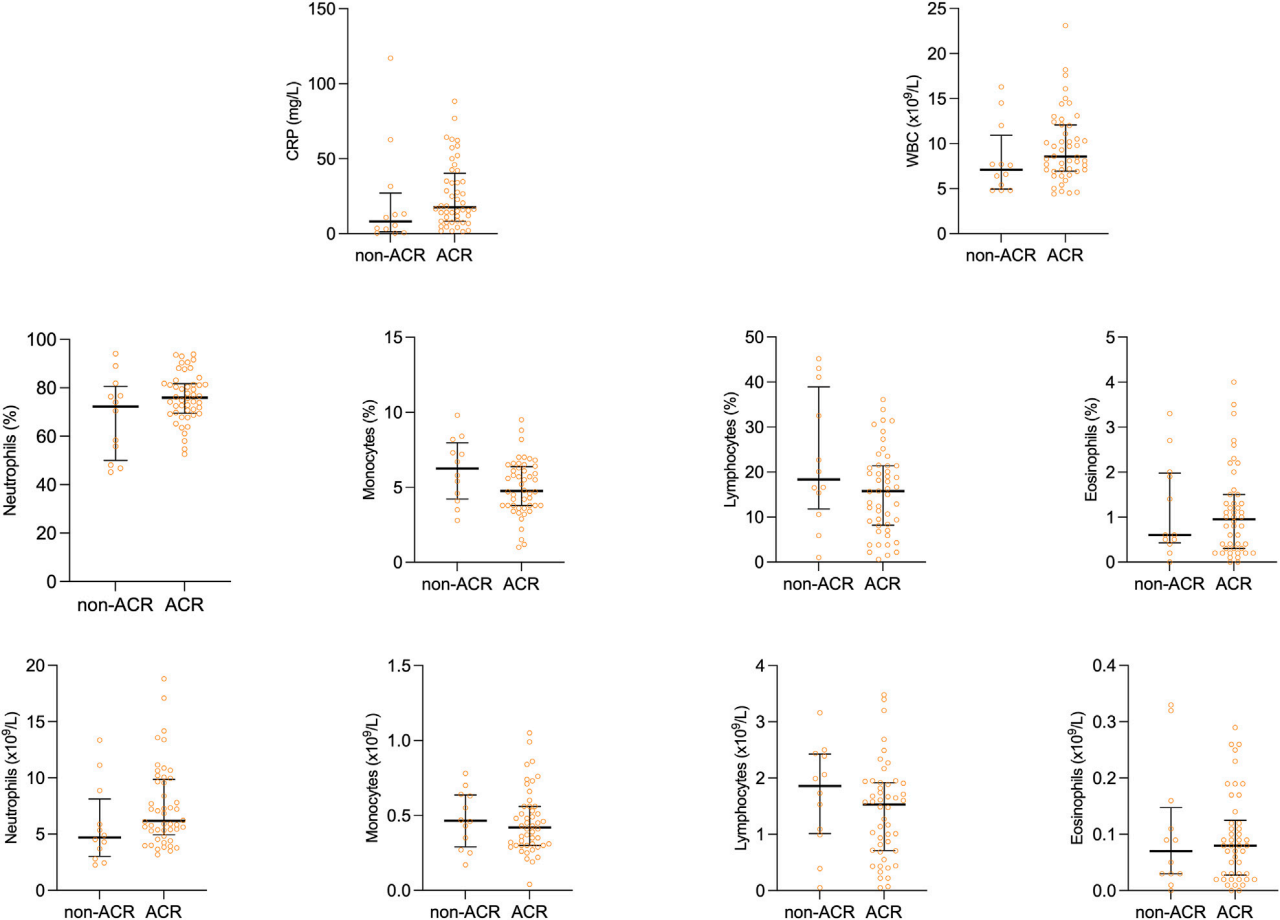
Scatter plots of peripheral blood cell profile comparing rejection group to control group. Median and interquartile range is visualized.

Our study cohort was initially stratified into two subgroups based on the presence or absence of ACR. These subgroups were subsequently compared in relation to differential white blood cell counts (WBC) and acute-phase proteins, specifically C-reactive protein (CRP). Figure 1 shows scatter plots with median and interquartile range of the measured values. Interestingly, no difference was observed in total WBC count, and percentages and counts of neutrophils, monocytes, lymphocytes, and eosinophils (Table 4), suggesting the limited efficacy of basic leukocyte parameters in predicting ACR within this context.

**TABLE 4:** Peripheral blood cell profile.

	no ACR (A0) n = 12	ACR (A1-3) n = 48	*p*-value
CRP (mg/L)	8.15 (2.4–17.8)	17.5 (8.45–36.83)	0.06
WBC count (×10^9^/L)	7.1 (5.25–8.78)	8.55 (7.05–12.03)	0.15
Neutrophils (%)	72.25 (53.95–78)	75.95 (69.70–81.50)	0.19
Neutrophils (×10^9^/L)	4.70 (3.48–6.63)	6.17 (5.17–9.73)	0.07
Monocytes (%)	6.25 (4.48–7.53)	4.75 (3.8–6.33)	0.09
Monocytes (×10^9^/L)	0.46 (0.33–0.63)	0.42 (0.30–0.56)	0.70
Lymphocytes (%)	18.35 (14.20–34.65)	15.75 (8.33–21.4)	0.18
Lymphocytes (×10^9^/L)	1.86 (1.06–2.40)	1.53 (0.72–1.91)	0.25
Eosinophils (%)	0.6 (0.48–1.93)	0.95 (0.3–1.45)	0.83
Eosinophils (×10^9^/L)	0.07 (0.03–0.12)	0.08 (0.03–0.12)	0.97

Data are expressed as median (interquartile range). Abbreviations: CPR, C-reactive protein; WBC, white blood cells.

On the other hand, CRP, an acute-phase protein synthesized in the liver due to interleukin-6 secretion by macrophages and T-cells, displayed variations between the observed groups. As shown in Figure 1, peripheral blood levels of CRP tended to be lower in the non-ACR group [8.15 (2.4–17.8) as compared to the ACR group 17.5 (8.45–36.83) *p* = 0.055].

### Neither T-Cell Subsets Nor B-Cells and Macrophages Exhibited Significant Elevation in Patients With Acute Cellular Rejection

**FIGURE 2:**
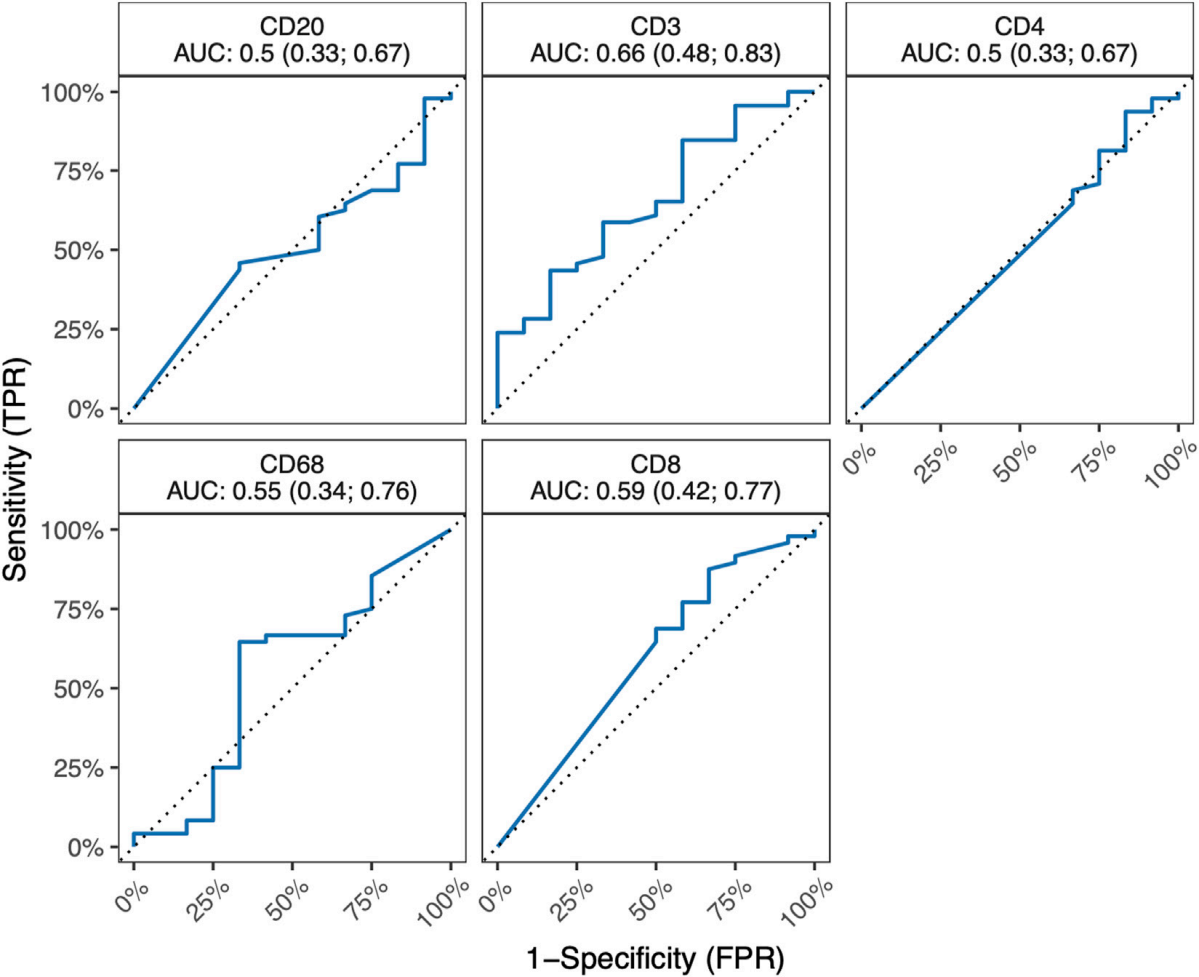
ROC curves for IHC markers CD3, CD4, CD8, CD20 a CD68, along with corresponding AUC values and their 95% confidence interval. All 95% confidence intervals include 0.5, which shows that none of the markers are good predictors.

In the course of our investigations, our primary objective revolved around elucidating the potential impact of ACR on the proportions of critical immune cell types (T-cells, B-cells, and macrophages) and demonstrating whether ACR elicits substantial changes in the abundance or distribution of these cell populations. Table 5 outlines the counts of positive immune cells per 1 mm^2^ for selected IHC markers. We hypothesized that examining the specific surface markers, such as CD3, CD4, CD8, CD20, and CD68, might offer a viable means of detecting the initial stages of ACR. Unfortunately, these markers did not show any differences between the ACR and non-ACR groups. As depicted in Figure 2, neither T-cell subsets nor B-cells and macrophages exhibited significant elevation in patients with ACR.

**TABLE 5:** Positive immune cell counts per 1 mm^2^ in lung tissue were determined for specific IHC markers in the study groups, excluding endothelial cells in PECAM-1 from the scoring system.

	no ACR (A0) n = 12	ACR (A1-3) n = 48	*p*-value
CD3	82 (38.5–97.75)	94.5 (56–136)	0.10
CD4	0 (0–5.25)	0 (0–6)	0.99
CD8	4.5 (0–30.25)	0 (0–19)	0.25
CD20	2 (0–10.25)	2.5 (0–18)	0.98
PD1	14 (6.5–42)	32 (19.75–59.75)	0.12
PD-L1	1.5 (0–5.25)	9.5 (3.25–18)	**0.0023**
CD68	37.5 (20.25–102.75)	77 (23.75–92.50)	0.61
PECAM-1	36.5 (35.25–43.25)	58 (40.75–84)	**0.0131**
CD47	275.5 (202–381.5)	338.5 (235.5–418)	0.29

Data are expressed as median (interquartile range). Two samples for PD-L1 were missing in both groups, and one sample for PD-1 was missing in the non-ACR group.

Bold values indicate statistical significance at the level of *p* < 0.05.

### PD-L1 Is Significantly Increased in Lung Transplant Recipients Exhibiting Acute Cellular Rejection

In the subsequent array of analyses, we examined immune checkpoints in lung tissue samples to understand the balance of activation/inhibition signals transmitted through immune receptors. Our primary focus was on the most prominent immune checkpoint pathway, predominantly occurring in T-cells, which involves the interaction between PD-1 and PD-L1 [33]. Following this, our attention shifted to exploring the novel potent macrophage checkpoint CD47, known as the “don’t eat me” signal [34]. While CD47 displayed no significant variations between the ACR and non-ACR group, striking differences were observed when analyzing the PD-L1 expression within lung allografts.

**FIGURE 3:**
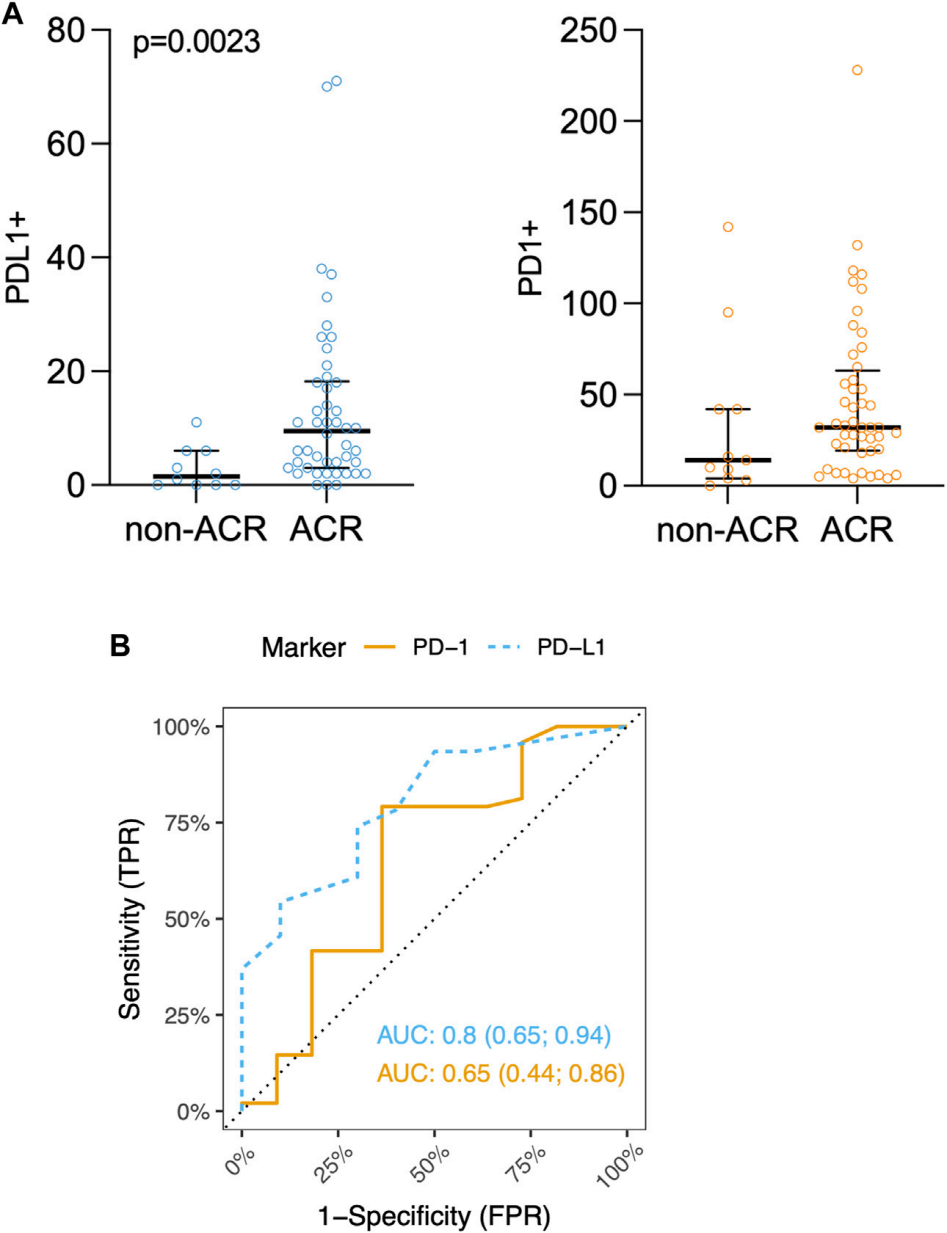
**(A)** Scatter plots of PD-L1+ and PD-1+ immune cells counts (positive immune cells per 1 mm^2^) in lung tissue biopsy. Median and interquartile range is visualized. **(B)** ROC curves for IHC markers PD-L1 and PD-1. Area under ROC-curves (AUC) (and associated 95% confidence interval) based on marked values for PD-L1 is 0.80 (0.65; 0.94) and for PD-1 is 0.65 (0.44; 0.86).

As shown in Figure 3A, PD-L1 exhibited significant increase in the rejection group (PD-L1 *p* = 0.0023), indicating an ongoing attempt to inhibit immune responses, particularly those involving T-cells. On the other hand, while the increase in PD-L1 levels might imply an effort to foster peripheral immune tolerance through its interaction with the PD-1 receptor, there was no observed increase in PD-1 receptor within the ACR cohort when compared to the non-ACR group. Figure 3B shows areas under ROC curves, and associated 95% confidence intervals, based on marked values for PD-L1 (0.80 confidence interval [0.65; 0.94]) and PD-1 (0.65 confidence interval [0.44; 0.86]). PD-L1 does not include 0.5 in its 95% confidence interval, therefore it can be a good ACR predictor. Supplementary Figure S1 shows Youden’s Index and Euclidian distance for PD-L1. Table 6, 7 show confusion matrices for PD-L1. PD-L1 remained significant (*p* = 0.0112) when analyzed across all ACR A grades collectively, as shown in Supplementary Table S5.

**TABLE 6:** Confusion matrix showing Youden’s Index for PD-L1 with cut-off value of 7 positive cells/mm^2^.

PD-L1		
Actual	Predicted	
	Rejected	Non-rejected
Rejected	25	21
Non-rejected	1	9

**TABLE 7:** Confusion matrix showing Euclidian distance for PD-L1 with cut-off value of 4 positive cells/mm^2^.

PD-L1		
Actual	Predicted	
	Rejected	Non-rejected
Rejected	34	12
Non-rejected	3	7

### In Human Lung Allografts, Leukocytes Exhibit Dynamic Transendothelial Migration

**FIGURE 4:**
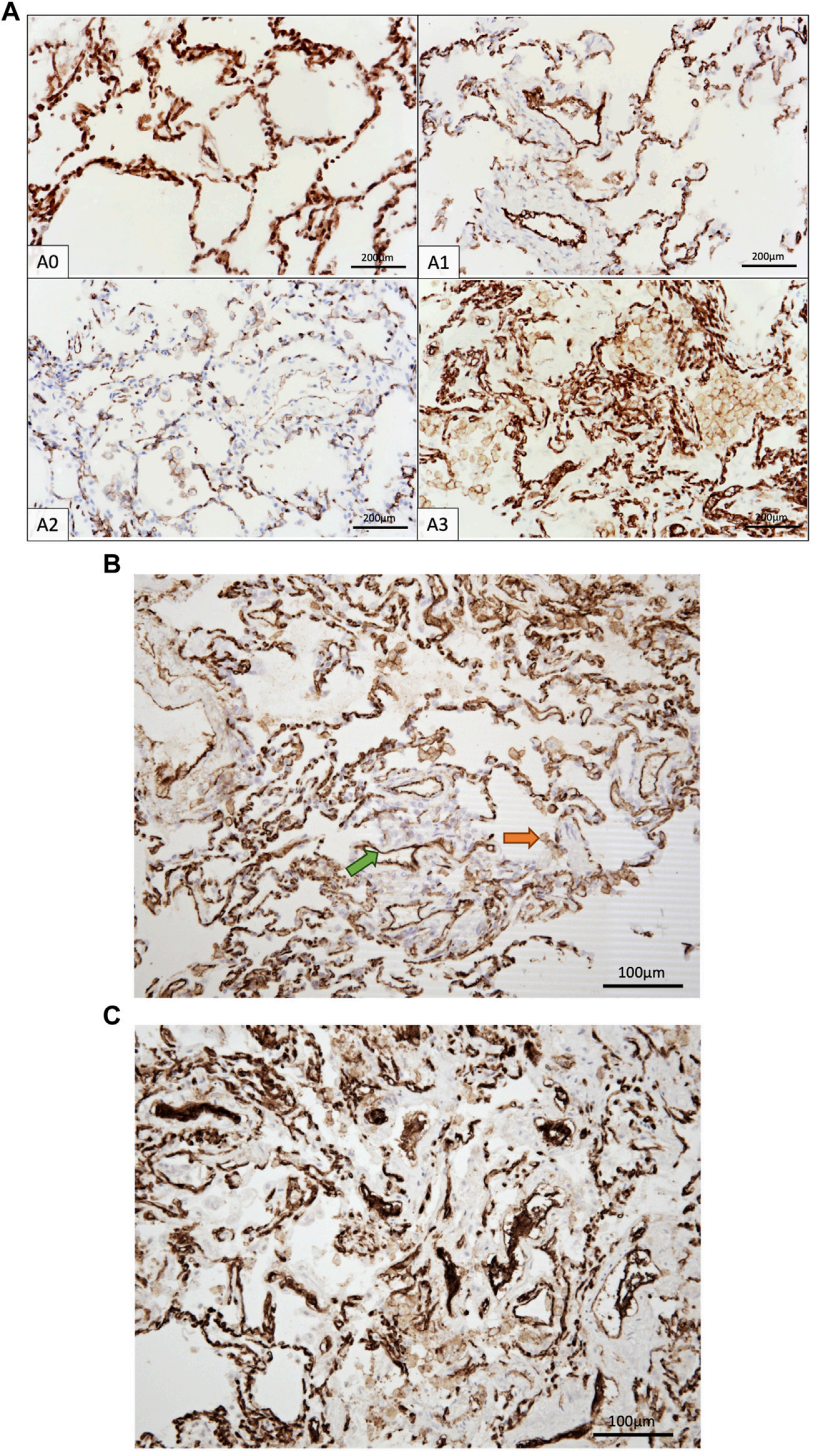
**(A)** IHC staining of CD31^+^ cells in control group and A1-A3 rejection groups. **(B)** CD31^+^ immune cells (orange arrow) and endothelia cells (green arrow). Only immune cells were counted. **(C)** Endothelium exhibiting CD31 positivity, presumably indicative of endothelial swelling associated with endothelitis, a characteristic frequently observed in A3.

PECAM-1, also known as CD31, plays a crucial role in facilitating the movement of leukocytes across the intercellular junctions of vascular endothelial cells during the process of leukocyte transmigration [35]. Given the increased scientifical interest in anti-PECAM-1 therapies blocking transendothelial migration of leukocytes, our aim was to investigate the potential involvement of PECAM-1 in ACR of lung allografts. Figures 4A–C show the PECAM-1 IHC staining of the samples.

**FIGURE 5:**
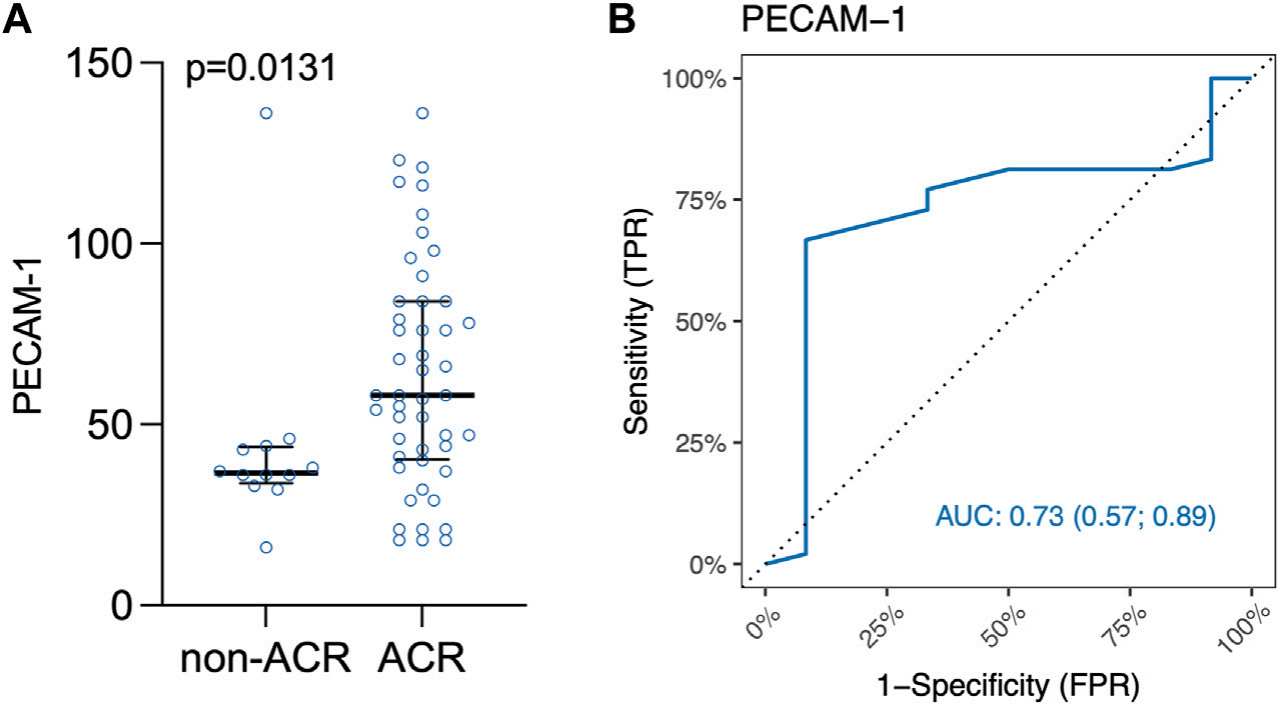
**(A)** Scatter plot of PECAM-1/CD31 immune cells in lung tissue biopsy. Median and interquartile range is visualized. **(B)** ROC curve for IHC marker PECAM-1. Area under ROC curve based on marked values for PECAM-1 is 0.73.

Interestingly, PECAM-1 expression, assessed via IHC, demonstrated a trend towards significance (*p* = 0.0874) when analyzed across all ACR A grades collectively (Supplementary Table S5). PECAM-1 was significantly elevated in LuTx patients diagnosed with ACR (*p* = 0.0131) compared to those without ACR (Figure 5A). This finding suggests that PECAM-1 may have promising potential as a biomarker for ACR detection.Figure 5B shows area under ROC curve based on marked value for PECAM-1 (0.73).

Both Youden’s Index and Euclidian distance cut-off point value based on the ranked values for PECAM-1 was 47 positive immune cells/mm^2^ (Supplementary Figure S2) indicating a threshold for distinguishing between samples that are positive or negative for PECAM-1 expression. Table 8 shows confusion matrix for PECAM-1.

**TABLE 8:** Confusion matrix showing Youden’s Index and Euclidian distance for PECAM-1/CD31 with cut-off value of 47 positive cells/mm^2^.

PECAM-1		
Actual	Predicted	
	Rejected	Non-rejected
Rejected	32	16
Non-rejected	1	11

### T Cell Functional Capacities Were Unaffected in the Rejection Group

To gain a deeper understanding of T cell functionality in LuTx, we conducted a multiplex bead-based immunoassay using Luminex technology on serum samples to evaluate key molecules reflecting T cytotoxic and proliferative function. These molecules included PD-L1, IL-2, Granzyme B, TNFα, and IFNγ.

This multi-faceted approach allowed us to assess the cytotoxic capacities of T cells, and the inflammatory environment in the context of graft survival.

In the rejection group, our analysis revealed an increase in serum levels of IL-2, a cytokine that plays a critical role in T cell proliferation and immune regulation, alongside a decrease in PD-L1 levels. However, these changes did not reach statistical significance, with *p*-values of 0.8046 for IL-2 and 0.1224 for PD-L1, respectively. This suggests that while there may be a trend in these biomarkers, the observed variations are not strong enough to draw definitive conclusions about their roles in rejection processes.

**FIGURE 6:**
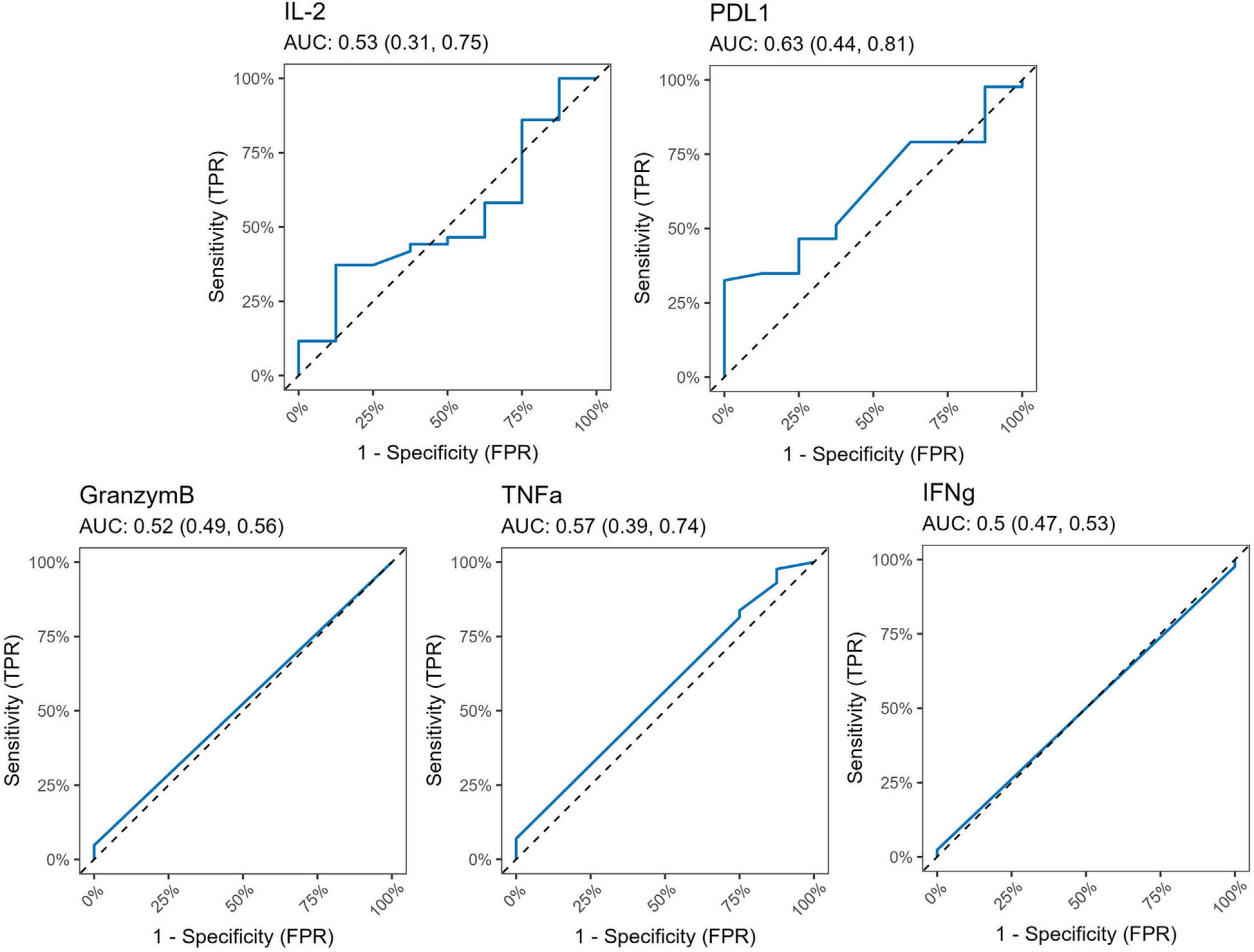
ROC curves for Luminex markers PD-L1, IL-2, Granzym B, TNFα and IFNγ, along with corresponding AUC values and their 95% confidence interval. All 95% confidence intervals include 0.5, which shows that none of the markers are good predictors.

Furthermore, no significant differences were detected in the levels of granzyme B, TNFα, and IFNγ, indicating that these immune markers may not be associated with rejection in this study population. Figure 6 shows areas under ROC curves, and associated 95% confidence intervals, based on marked values for analyzed molecules. All 95% confidence intervals include 0.5, which shows that none of the markers are good predictors.

## Discussion

LuTx patients frequently face ACR complications, impacting lung function and contributing to CLAD. Our study analyzed ACR and non-ACR groups, focusing on variations in WBC counts and acute-phase proteins. Since these variables may be affected by immunosuppressive treatment, our study included only patients treated at our center, where the standard maintenance therapy consists of tacrolimus, mycophenolate, and corticosteroids. The induction immunosuppressive regimens varied among individual patients; however, there were no statistically significant differences between the ACR and non-ACR groups. Similar investigations by Vos et al. linked systemic inflammation, CRP levels, and graft failure, aligning with elevated CRP during acute heart rejection observed by Eisenberg et al [36–38]. In our study, despite no significant differences in total WBC count or cell percentages, CRP levels tended to be higher in the ACR group, indicating a potential association. Although the infection status of our patients did not statistically differ between ACR and non-ACR groups, changes in CRP levels should always be interpreted with caution, as not only accompanying infections, but also stress, inflammatory conditions, and other factors, may influence CRP [39, 40].

Next, we employed IHC to assess specific surface markers, aiming to uncover how ACR might influence the ratios of crucial immune cell (T-cells, B-cells, macrophages). Unfortunately, no significant differences were found between ACR and non-ACR groups.

Upon examining checkpoint molecules, it became apparent that CD47 did not seem a feasible marker for ACR. However, our focus shifted towards exploring the PD-1 and PD-L1 inhibitory pathway. To date, several studies have explored the functions of PD-1 and PD-L1 in transplantation. Wang et al. underscored their vital role in establishing cardiac allograft tolerance in mouse models [41]. Tanaka et al. highlighted PD-L1’s pivotal role in both inducing and maintaining peripheral tolerance following heart transplantation by modulating the equilibrium among T-cell subsets [42]. Additionally, Choudhary et al. observed an upregulation of PD-L1 within cardiomyocytes, demonstrating a correlation with the severity of ACR after transplantation [43].

Righi et al., focusing on 24 LuTx patients, revealed the importance of PD-1 in acute rejection and its progression into CLAD. They proposed evaluating PD-1-expressing lymphocytes in transbronchial biopsies for prognostic monitoring [44]. Subsequently, Kaiho et al. investigated PD-1/PD-L1 in acute rejection using a mouse tracheal transplantation model, finding a PD-L1-mediated immune checkpoint association with rejection, suggesting a potential immunotherapy target in LuTx [45].

Our study contributes to the understanding of the involvement of the PD-1/PD-L1 axis in LuTx by demonstrating a significant increase of tissue PD-L1 levels within the ACR group. While it remains uncertain which cells produce PD-L1 in lung allografts *in vivo*, this increase indicates an active effort to suppress immune responses, particularly those associated with T-cells. However, we did not detect a concurrent rise in the PD-1 receptor among the ACR cohort when compared to the non-ACR group.

We hypothesize that this phenomenon may result from PD-L1 production by various non-immune cells within the lung tissue, such as epithelial and endothelial cells, representing a localized immune suppression effort within the graft, primarily mediated by tissue-specific responses rather than T-cell-mediated modulation [46]. Furthermore, animal transplant models have shown that blocking PD-L1 leads to rejection, while blocking PD-1 and PD-L2 has no effect on graft survival. This indicates that PD-L1 and PD-L2 may play distinct roles in promoting tolerance, with PD-L1 expression, rather than PD-1 expression, emerging as the more reliable marker of immune regulation in transplantation [20].

Thus, the role of the PD-1/PD-L1 mechanism in acute rejection after lung transplantation has not yet been elucidated. These data, in accordance with previous studies, may imply the impairment of peripheral tolerance in LuTx recipients experiencing ACR.

In contemporary oncology, checkpoint molecules have emerged as pivotal targets in the therapeutic landscape, particularly within the realm of cancer treatment. This prominence arises from their capacity to modulate immune responses, a feature notably exploited to counteract the immunosuppressive microenvironment characteristics of malignancies [47]. Conversely, in the context of transplantation, the immune system often experiences heightened activation, resulting in the potential rejection of the transplanted organ. Hence, the contrasting immunological dynamics observed between cancer and transplantation underscore the likelihood of checkpoint molecules assuming a significant role in the latter scenario as well.

Khan et al. showed that the CTLA4, combined with the Fc portion of human immunoglobulin G1 (CTLA4-Ig) used as monotherapy immunosuppressant in mouse airway transplants promoted a favorable phase of immunotolerance, which facilitated microvascular and tissue repair [48].

The TIM family, notably TIM-1 and TIM-3, are pivotal regulators of the immune response and have been investigated in experimental transplant models. Murine studies reveal that inhibiting TIM-1 and boosting TIM-3 signaling enhances allograft outcomes [49]. The consistent findings across acute and chronic rejection models underscore the potential of TIM-3 interaction in mitigating detrimental immune responses [49]. The administration of stable galectin-9 in murine skin and cardiac transplants prolongs allograft survival by decreasing Th1 and Th17 cytokines and fostering Tregs [50–52]. To date, studies evaluating the role of LAG-3 in lung transplantation are lacking.

While there is speculation regarding the therapeutic utility of checkpoint molecules in modulating immune responses to prevent rejection, it is imperative to consider the potential adverse effects associated with such therapies. Of particular concern is the development of autoimmunity, a consequence that is undesirable across various clinical context. Moreover, according to Cui et al., immune checkpoint inhibitors were significantly associated with rejection in solid organ transplant recipients [53]. Therefore, any exploration of checkpoint inhibitor therapy in transplantation must carefully weigh the benefits of immune modulation against the risk of inducing autoimmune phenomena.

PECAM-1 plays a pivotal role in facilitating the migration of leukocytes across intercellular junctions within vascular endothelial cells during the transmigration process [35, 54]. The protective role of PECAM-1 in acute rejection has been demonstrated in various studies, yet its expression has not been previously analyzed by IHC in lung tissue during rejection episodes [55]. In 2022, Tran-Dinh et al. introduced an AI model evaluating CD31 cleavage for early ACR detection post LuTx [55]. We assessed the immunohistochemical surface expression of PECAM-1 in leukocytes from two distinct groups of LuTx recipients: individuals experiencing ACR and those without such complications. To our surprise, LuTx patients diagnosed with ACR showed a significant increase in PECAM-1 expression (IHC), prompting us to hypothesize that inhibiting transendothelial migration might represent a therapeutic approach for ACR.

In oncology, endothelial-immune cell interactions within the tumor microenvironment influence immune infiltration and function, highlighting the critical role of endothelial cells in immune response [56]. There is no reason to believe this would be any different in transplantation. Notably, endothelial cells in the donor lung are among the first to encounter the recipient’s immune system.

PECAM-1 is involved in a wide array of processes related to inflammation, vascular biology, and various immune functions [57]. It has several splice variants, each capable of exhibiting distinct adhesive properties, which may subsequently impact its ligand-binding characteristics and functional role in leukocyte transmigration [58, 59]. The functional role of PECAM-1 is influenced by multiple factors, including the nature and tissue localization of the inflammatory response, as well as genetic determinants [57].

PECAM-1 possess both pro- and anti-inflammatory roles. Besides facilitating transendothelial leukocyte migration, it also plays a role in dampening leukocyte activation and reducing pro-inflammatory cytokine production [60]. In the context of ACR, macrophages expressing PECAM-1 may polarize into M2 subset which exhibit anti-inflammatory and graft-protective effects [61]. Thus, high PECAM-1 expression in the graft may reflect immune cell infiltration as well as active repair processes and endothelial resilience.

This study shows the potential of IHC in ACR diagnosis. Despite the additional cost, time, and effort required to perform IHC, its application could be advantageous in borderline cases as a supplementary technique to traditional histopathology. To improve assessments, the ISHLT recommends obtaining at least five adequate samples, reducing variability [32]. This is particularly crucial in cases of ACR, especially when confronting lower-grade rejection. This variability not only presents challenges in individual patient management but also hinders efforts to achieve standardization in multicenter trials [11, 62]. Identifying appropriate IHC markers could help tackle these issues and our data suggests that both PD-L1 and PECAM-1 need further exploration in ACR.

In order to better understand T cell functionality in LuTx, we also employed a multiplex bead-based immunoassay using Luminex technology to assess key molecules that reflect T cell cytotoxicity and proliferation. The molecules analyzed were PD-L1, IL-2, Granzyme B, TNFα, and IFNγ. This approach aimed to provide insights into the cytotoxic potential of T cells and the surrounding inflammatory environment, which are crucial for improving graft survival, formulating targeted therapies, and enhancing outcomes for transplant patients.

The role of IL-2 in acute lung rejection has been previously reported [63–65]. Luminex analysis in our study cohort did not yield significant results. However, elevated levels of IL-2 in patients with ACR is in line with the older work of Jordan et al. [66] Our Luminex analyses were constrained by the small size of the study cohort. To this we also attribute the inconclusive results of other biomarkers investigated by the Luminex method. Future studies with larger sample sizes and the inclusion of more relevant biomarkers, such as PECAM-1, could provide more insightful findings.

In our center, cryobiopsies are the standard of care. The Zurich group has demonstrated that cryobiopsies offer a superior diagnostic yield for ACR compared to forceps biopsies, leading to reclassification and treatment strategy changes in 28.6% of cases [67]. Our findings show that nearly half of the samples in this cohort exhibit an A1 rejection grade. Notably, identifying A1 rejection in a clinically stable patient through biopsy may not necessitate therapeutic intervention.

This study adopts a retrospective design, encompassing solely double lung transplant patients with samples available in archives in a single high-volume transplant center. Observational design, limited cohort size and group size imbalances are notable. However, the cohorts, aside from size differences, exhibit consistent characteristics. Our selection might indeed introduce bias and therefore, a prospective study would be imperative to also ascertain an accurate representation of the prevalence within our patient cohort. Despite widespread use of IHC, interpretational variability also remains a challenge.

Conducting larger studies is essential for evaluating immunohistochemistry (IHC), with a specific focus on PD-L1/PECAM-1 markers, in the diagnosis of ACR.

## Conclusion

IHC investigations of PECAM-1 and PD-L1 markers might be valuable for diagnosing ACR. Further research is required to enhance our understanding of the role of immune checkpoint inhibitors in lung transplantation.

## Data Availability

The raw data supporting the conclusions of this article will be made available by the authors, without undue reservation.

## Abbreviations

ACR, acute cellular rejection; AMR, acute humoral rejection; APCs, antigen presenting cells; BOS, bronchiolitis obliterans syndrome; CLAD, chronic lung allograft dysfunction; CMV, cytomegalovirus; COPD, chronic obstructive pulmonary disease; CRP, C-reactive protein; DBD, donation after brain death; IHC, immunohistochemistry; IFNγ, interferon gamma; ILD, interstitial lung disease; ISHLT, the International Society for Heart and Lung Transplantation; PAH, pulmonary arterial hypertension; PD-1, programmed death-receptor 1; PD-L1, programmed death-ligand 1; LuTx, lung transplantation; RAS, restrictive allograft syndrome; ROC, receiver operating characteristics; TNFα, tumor necrosis factor alpha; WBC, white blood cells.
