# Supplementary material for: Elevated PD-L1 and PECAM-1 as Diagnostic Biomarkers of Acute Rejection in Lung Transplantation
Source: Transpl Int. 2024 Nov 21;37:13796. doi: 10.3389/ti.2024.13796 (PMC11617192; doi:10.3389/ti.2024.13796)
Supplement: Supplementary file 1 [file DataSheet2.docx]

**Supplementary Table S1:** Distribution of acute cellular rejection grades in the study cohort.

| A0 | BX | 5 |
| --- | --- | --- |
|  | B0 | 7 |
| A1 | BX | 14 |
|  | B0 | 9 |
|  | B1R | 6 |
| A2 | BX | 6 |
|  | B0 | 5 |
|  | B1R | 5 |
|  | B2R | 2 |
| A3 | B1R | 1 |

**Supplementary Table S2:** Rates (percentages) of induction immunosuppression and plasmapheresis in study groups. Tacrolimus, mycophenolate and corticosteroids are not included in the table.

|  | **no ACR (A0) n = 12** | **ACR (A1-3) n = 48** |
| --- | --- | --- |
| Basiliximab | 92% | 90% |
| Rabbit anti-thymocyte globulin | 0% | 16% |
| Plasmapheresis | 8% | 13% |
| Alemtuzumab | 8% | 13% |

**Supplementary Table S3:** Rates (percentages) of lung infections in study groups at the time of cryobiopsy. ACR = acute cellular rejection.

|  | **no ACR (A0) n = 12** | **ACR (A1-3) n = 48** |
| --- | --- | --- |
| Bacterial | 33% | 31% |
| Viral | 0% | 10% |
| Fungal | 16% | 19% |

**Supplementary Table S4:** Distribution of underlying immunological conditions in study groups (excluding primary diseases for which transplantation was performed). *Abbreviations: ANCA - anti-neutrophil cytoplasmic antibodies, ANA - antinuclear antibody, IgG - immunoglobulin G*

|  | **no ACR (A0) n = 12** | **ACR (A1-3) n = 48** |
| --- | --- | --- |
| Polyvalent allergy | 0 | 2 |
| Eosinophilia | 0 | 2 |
| Hyperimmunoglobulinemia with positive ANCA | 0 | 1 |
| Celiac disease | 1 | 0 |
| Psoriasis | 1 | 0 |
| Psoriatic artritis | 0 | 1 |
| Positive ANA | 0 | 1 |
| Rheumatoid arthritis | 0 | 1 |
| Hypogammaglobulinemia | 0 | 1 |
| Dysgammaglobulinemia type III | 0 | 1 |
| Antiphospholipid antibody positivity | 0 | 1 |
| Immunodeficiency with low IgG | 0 | 1 |
| Hypereosinophilic syndrome | 0 | 1 |
| Ankylosing spondylitis | 0 | 1 |

**Supplementary Table S5:** Distribution of PD-L1 and PECAM-1 immune cell counts per 1 mm^2^ across acute cellular rejection grades (A0 to A3), excluding endothelial cells in CD31 from the scoring system. Data are expressed as median (interquartile range). For PD-L1, only 10 samples were available in A0, and one sample was missing in A2.

|  | **A0 (n=12)** | **A1 (n=29)** | **A2 (n=18)** | **A3 (n=1)** | **p-value** |
| --- | --- | --- | --- | --- | --- |
| PD-L1 | 2 (0-5) | 7 (4-26) | 11 (2-14) | N/A | **0.0112** |
| PECAM-1 | 37 (35-43) | 69 (44-84) | 54 (39-68) | 65 | 0.0874 |
