# Supplementary material for: Elevated PD-L1 and PECAM-1 as Diagnostic Biomarkers of Acute Rejection in Lung Transplantation
Source: Transpl Int. 2024 Nov 21;37:13796. doi: 10.3389/ti.2024.13796 (PMC11617192; doi:10.3389/ti.2024.13796)
Supplement: Supplementary file 2 [file DataSheet1.docx]

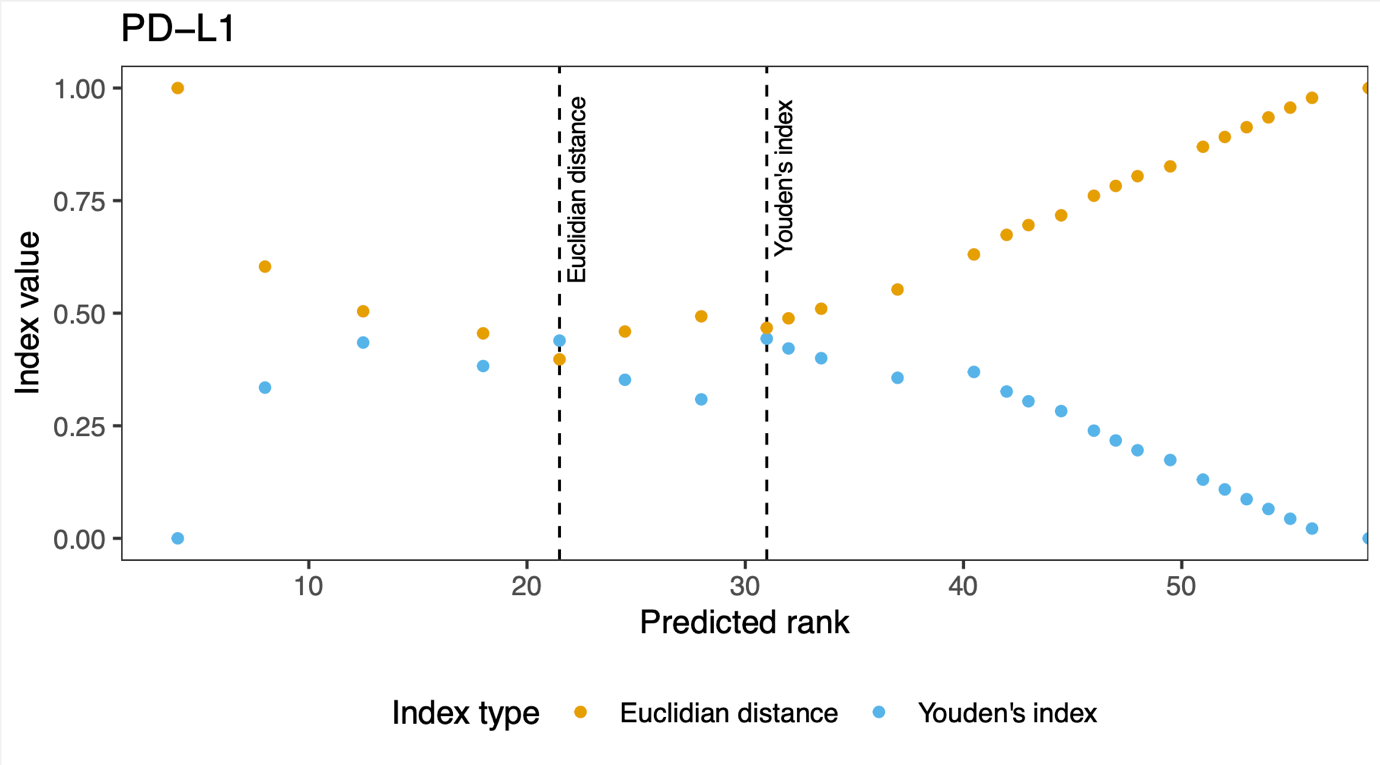


**Supplementary Figure S1:** Youden’s index and the Euclidian distance for PD-L1.

**
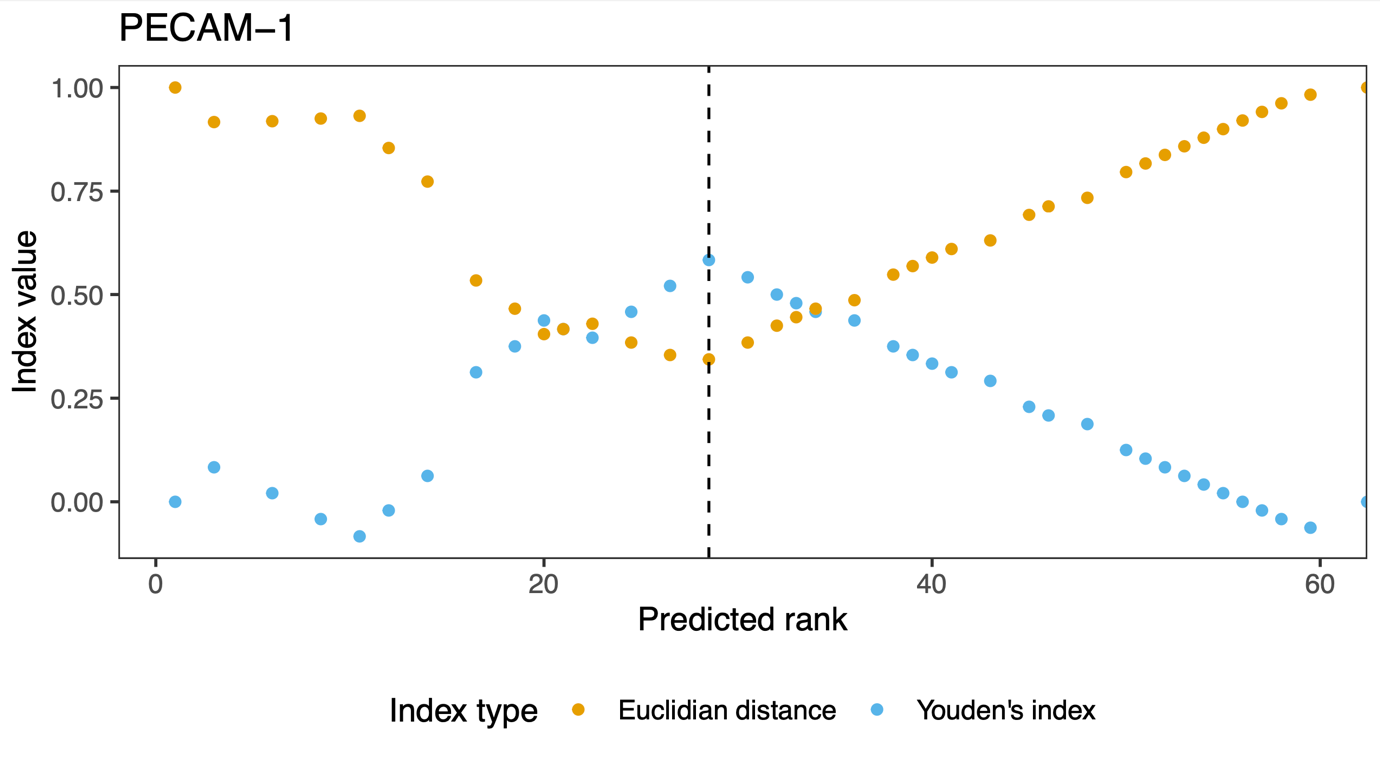
**

**Supplementary Figure S2:** Youden’s index and the Euclidian distance for PECAM-1/CD31.
